# Embryonic stem cell-derived extracellular vesicle-mimetic nanovesicles rescue erectile function by enhancing penile neurovascular regeneration in the streptozotocin-induced diabetic mouse

**DOI:** 10.1038/s41598-019-54431-4

**Published:** 2019-12-27

**Authors:** Mi-Hye Kwon, Kang-Moon Song, Anita Limanjaya, Min-Ji Choi, Kalyan Ghatak, Nhat Minh Nguyen, Jiyeon Ock, Guo Nan Yin, Ju-Hee Kang, Man Ryul Lee, Yong Song Gho, Ji-Kan Ryu, Jun-Kyu Suh

**Affiliations:** 1 National Research Center for Sexual Medicine and Department of Urology, Inha University School of Medicine, Incheon, 22332 Korea; 2 Department of Pharmacology and Medicinal Toxicology Research Center, Inha University School of Medicine, Incheon, 22212 Korea; 3 Soonchunhyang Institute of Medi-bio Science (SIMS) and Institute of Tissue Regeneration, College of Medicine, Soon Chun Hyang University, Cheonan-si, Chungcheongnam-do 31151 Korea; 4 Department of Life Sciences, Pohang University of Science and Technology, Pohang, Kyeongsangbuk-do 37673 Korea

**Keywords:** Extracellular signalling molecules, Urological manifestations

## Abstract

Extracellular vesicles (EVs) have attracted particular interest in various fields of biology and medicine. However, one of the major hurdles in the clinical application of EV-based therapy is their low production yield. We recently developed cell-derived EV-mimetic nanovesicles (NVs) by extruding cells serially through filters with diminishing pore sizes (10, 5, and 1 μm). Here, we demonstrate in diabetic mice that embryonic stem cell (ESC)-derived EV-mimetic NVs (ESC-NVs) completely restore erectile function (~96% of control values) through enhanced penile angiogenesis and neural regeneration *in vivo*, whereas ESC partially restores erectile function (~77% of control values). ESC-NVs promoted tube formation in primary cultured mouse cavernous endothelial cells and pericytes under high-glucose condition *in vitro*; and accelerated microvascular and neurite sprouting from aortic ring and major pelvic ganglion under high-glucose condition *ex vivo*, respectively. ESC-NVs enhanced the expression of angiogenic and neurotrophic factors (hepatocyte growth factor, angiopoietin-1, nerve growth factor, and neurotrophin-3), and activated cell survival and proliferative factors (Akt and ERK). Therefore, it will be a better strategy to use ESC-NVs than ESCs in patients with erectile dysfunction refractory to pharmacotherapy, although it remains to be solved for future clinical application of ESC.

## Introduction

Penile erection is an integrated neurovascular event between endothelial cells, mural cells (vascular smooth muscle cells and pericytes), and neuronal cells^1–3^. Erectile dysfunction (ED) is a highly prevalent among males with diabetes, affecting more than half of the men with this condition^4^. A variety of pathological conditions, including microvascular dysfunction, peripheral neuropathy, and hormonal disturbances, are responsible for diabetic ED^2^. Because the pharmacological efficacy of phosphodiesterase type 5 (PDE5) inhibitors depends on endogenous nitric oxide (NO) production, a lack of bioavailable NO in diabetic men as the results of severe penile neurovascular dysfunction is the most important reason for poor responsiveness to these drugs^5–7^. Therefore, the development of a new therapeutic strategy that regenerates damaged penile neurovascular structure is needed.

Extracellular vesicles (EVs), which include apoptotic bodies, micro vesicles (also called microparticles), and exosomes, have been known to play a crucial role in cell-cell communication in a variety of conditions^8,9^. EVs are nano-sized spherical bilayered proteolipids encasing various components^10^. EVs carry genetic components (mRNAs and miRNAs), lipids, and numerous proteins, which are fundamental to their biogenesis and cell type- or context-specific actions^11^.

It was demonstrated that EVs derived from a variety of cells, such as endothelial cells, endothelial colony-forming cells, and mesenchymal stem cells, can modulate angiogenesis^12^. EVs have also been suggested to play an important role in neuronal development and neuroprotection^13,14^. Schwann cell-derived EVs have an ability to enhance neurite outgrowth^15^. The use of EVs has advantages over their cells or stem cell of origin, avoiding malignant transformation, immune rejection, and difficulties in cell manufacturing^16^.

Recent study has demonstrated in diabetic rats that intracavernous injection of adipose-derived EVs isolated by ultracentrifugation of culture supernatant restored erectile function by increasing cavernous endothelial content and by decreasing cavernous fibrosis. However, the recovery of erectile function induced by adipose-derived EVs was partial and did not reach the level found in normal age-matched controls^17^. Moreover, one of the major hurdles in the clinical application of EV-based therapy is the low production yield of EVs and the difficulty of purification^10^. To overcome these limitations, our colleagues recently developed cell-derived EV-mimetic nanovesicles (NVs) by extruding cells serially through filters with diminishing pore sizes (10, 5, and 1 μm). These cell-derived EV-mimetic NVs have similar characteristics with the natural EVs, but have 100-fold higher production yield^18,19^.

In the present study, therefore, we firstly determined the optimal dosage of embryonic stem cell (ESC)-derived EV-mimetic NVs (ESC-NVs) to induce maximal erectile function recovery in a mouse model of diabetic ED. We also directly compared the efficacy of ESC-NVs with ESC in terms of erectile function recovery. And then, we examined the proangiogenic or neurotrophic effects of ESC-NVs in primary cultured mouse cavernous endothelial cells (MCEC) and pericytes (MCP) *in vitro*; in cultured aortic ring and major pelvic ganglion (MPG) *ex vivo*; and in diabetic mice *in vivo*.

## Results

### Preparation and characterization of ESC-NVs

**Figure 1:**
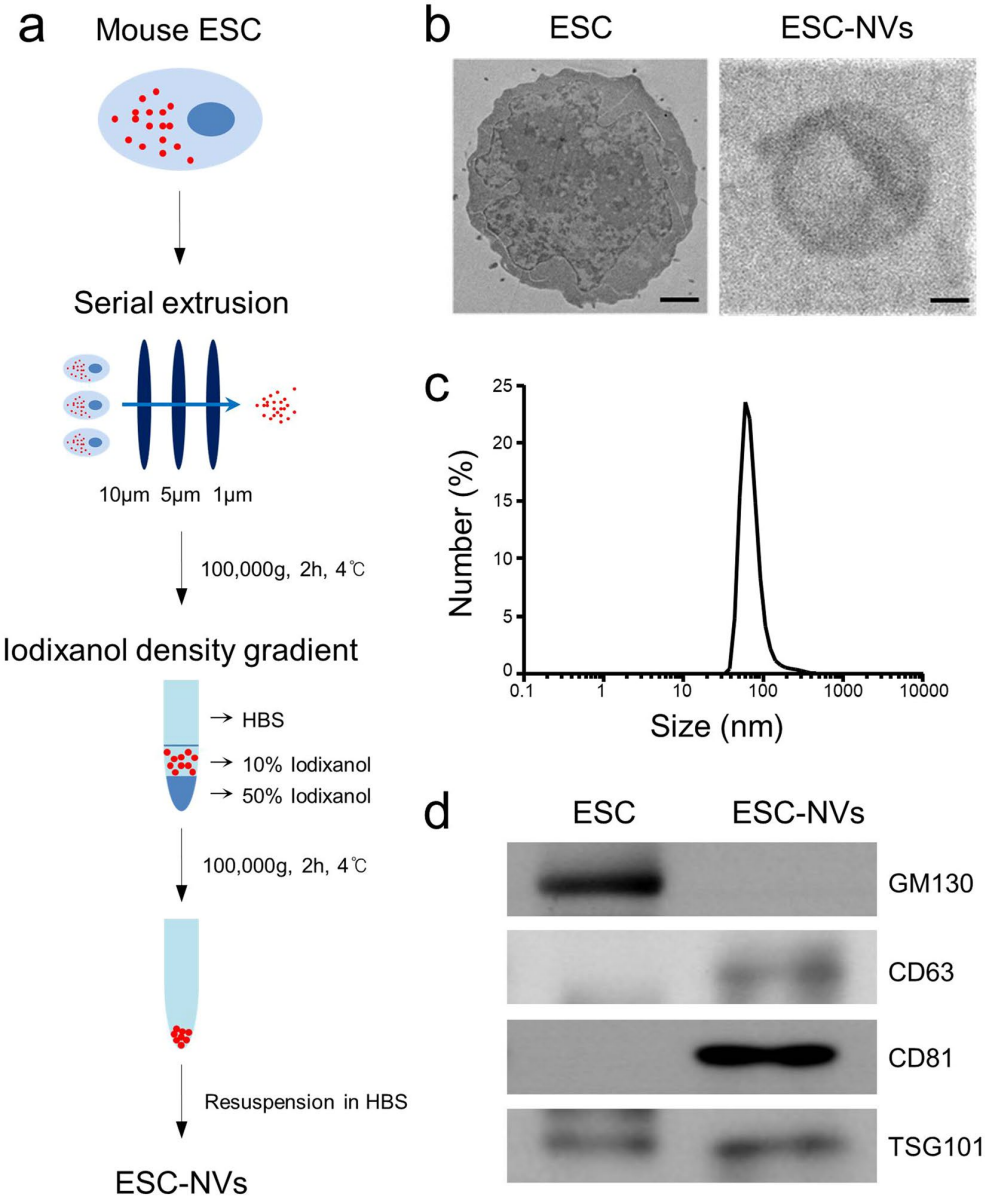
Preparation and characterization of embryonic stem cell (ESC)-derived extracellular vesicle-mimetic nanovesicles (ESC-NVs). (**a)** Schematic diagram of the experimental procedure for preparation of ESC-NVs. **(b)** Representative transmission electron micrograph images of ESC and ESC-NVs. Scale bars = 1000 nm (ESC) or 25 nm (ESC-NVs). **(c)** Size distribution of ESC-NVs measured by dynamic light scattering analysis. **(d)** Representative Western blot for a negative marker for NVs (GM130) or positive markers for NVs (CD63, CD81, and TSG101). HBS, HEPES (﻿4-(2-hydroxyethyl)-1-piperazineethanesulfonic acid)-buffered saline.

EV-mimetic NVs were prepared from ESC according to the procedure described in Fig. 1a. Cryo-transmission electron microscopy of ESC-NVs showed closed vesicles devoid of the parent cells, cellular debris, and protein aggregates (Fig. 1b). A dynamic light scattering analysis demonstrated that the average diameter of the purified ESC-NVs was 74.7 ± 5.2 nm (Fig. 1c). This finding is similar to the results obtained by transmission electron micrograph images. The average number of ESC-NVs measured by nanoparticle tracking analysis was 13.9 × 10^8^ particles per 1 μg of total protein. Western blot analysis revealed that ESC-NVs expressed positive markers for exosomes, such as CD63, CD81, and TSG101. GM130, a peripheral membrane protein that is bound to the Golgi complex, was not detected in ESC-NVs, although donor ESC expressed GM130 (Fig. 1d).

### Metabolic variables

Body weight was significantly lower in the diabetic mice than that of control mice. In addition, diabetic group exhibited significantly increased fasting and postprandial blood glucose concentrations compared with the controls. No significant differences in body weight and blood glucose levels were found between the diabetic groups, regardless of the treatment given (Tables 1 and 2).

**Table 1:** Metabolic and physiologic parameters 2 weeks after treatment with embryonic stem cells (ESC).

	Control	STZ-induced diabetic mice	
		PBS	ESC (3 × 10^5^ cell/20 µL)
Body weight (g)	28.9 ± 0.8	22.3 ± 1.2*	23.3 ± 0.5*
Fasting glucose (mg/dL)	89.8 ± 4.0	247.6 ± 40.9*	224.8 ± 17.4*
Postprandial glucose (mg/dL)	161.2 ± 6.9	464.8 ± 20.2*	470.0 ± 20.3*
MSBP (mm Hg)	103.4 ± 1.6	110.7 ± 1.8	105.9 ± 2.2

Values are the mean ± SE from N = 5 animals per group. *P < 0.01 vs. control group. STZ, streptozotocin; MSBP, mean systolic blood pressure.

**Table 2:** Metabolic and physiologic parameters 2 weeks after treatment with embryonic stem cell (ESC)- derived extracellular vesicle-mimetic nanovesicles (ESC-NVs).

	Control	STZ-induced diabetic mice	
		HBS	ESC-NVs (1 µg/20 µL)
Body weight (g)	30.2 ± 0.3	24.5 ± 0.4*	24.3 ± 0.4*
Fasting glucose (mg/dL)	104.8 ± 6.9	232.0 ± 19.6*	247.0 ± 23.6*
Postprandial glucose (mg/dL)	159.4 ± 5.3	535.8 ± 25.7*	544.6 ± 22.0*
MSBP (mm Hg)	95.1 ± 0.6	103.1 ± 1.6	99.1 ± 2.2

Values are the mean ± SE from N = 5 animals per group. *P < 0.01 vs. control group. STZ, streptozotocin; MSBP, mean systolic blood pressure.

### ESC-NVs fully, and ESCs partially restore erectile function in the diabetic mice

**Figure 2:**
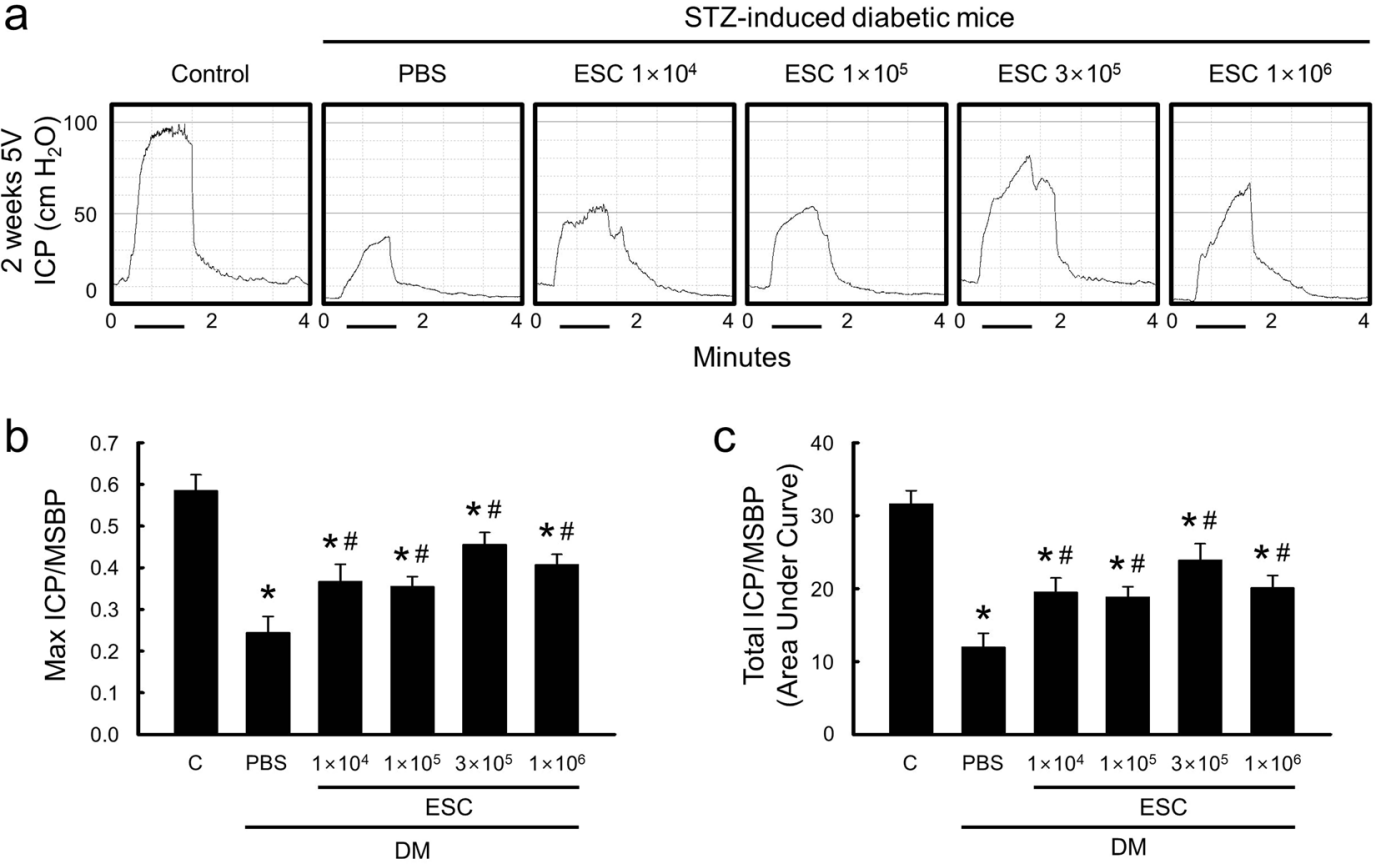
Embryonic stem cells (ESC) partially restore erectile function in the diabetic mice. (**a)** Representative intracavernous pressure (ICP) responses for the age-matched control (C) and diabetic mice stimulated at 2 weeks after intracavernous injections of PBS (days −3 and 0; 20 μL) or ESC (days -3 and 0; 1 × 10^4^, 1 × 10^5^, 3 × 10^5^, or 1 × 10^6^ cells/20 µL). The stimulus interval is indicated by a solid bar. **(b**,**c)** Ratios of mean maximal ICP and total ICP (area under the curve) to mean systolic blood pressure (MSBP) were calculated for each group. Each bar depicts the mean (±SE) values from N = 5 animals per group. *P < 0.01 vs. control group; ^#^*P* < 0.05 vs. PBS-treated diabetic group. DM, diabetes mellitus; PBS, phosphate-buffered saline; STZ, streptozotocin.

**Figure 3:**
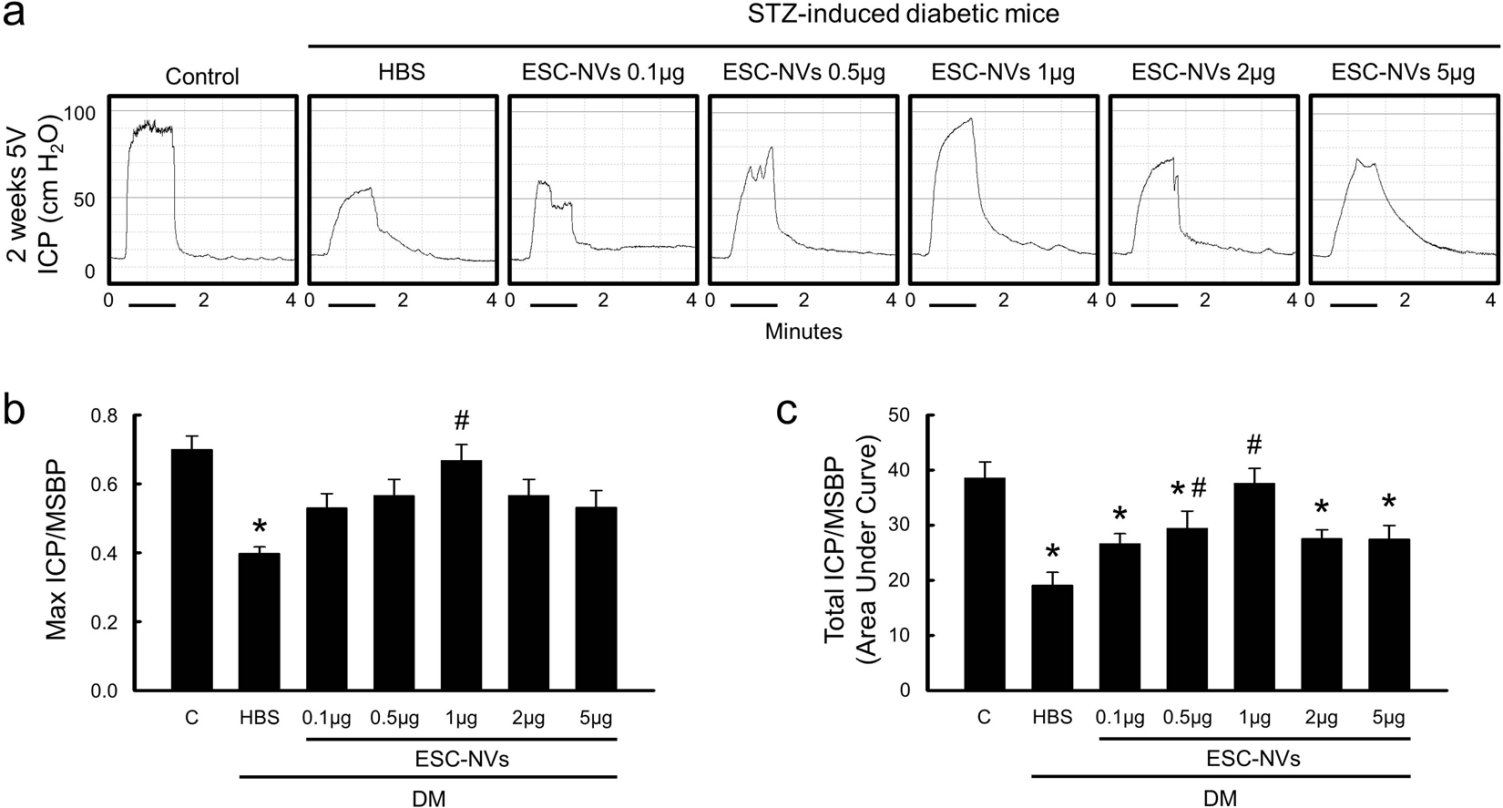
Embryonic stem cell (ESC)-derived extracellular vesicle-mimetic nanovesicles (ESC-NVs) completely restore erectile function in the diabetic mice. (**a)** Representative intracavernous pressure (ICP) responses for the age-matched control (C) and diabetic mice stimulated at 2 weeks after intracavernous injections of HBS (days -3 and 0; 20 μL) or ESC-NVs (days -3 and 0; 0.1 µg, 0.5 g, 1 µg, 2 µg, or 5 µg/20 µL). The stimulus interval is indicated by a solid bar. **(b**,**c)** Ratios of mean maximal ICP and total ICP (area under the curve) to mean systolic blood pressure (MSBP) were calculated for each group. Each bar depicts the mean (±SE) values from N = 5 animals per group. *P < 0.05 vs. control group; ^#^*P* < 0.05 vs. HBS-treated diabetic group. DM, diabetes mellitus; HBS, HEPES (4-(2-hydroxyethyl)-1-piperazineethanesulfonic acid)-buffered saline; STZ, streptozotocin.

To determine the physiological relevance of intracavernous injection of ESC or ESC-NVs, we performed nerve-induced erectile function study. Erectile function parameters, such as the ratios of maximal intracavernous pressure (ICP) and total ICP to mean systolic blood pressure (MSBP), were profoundly decreased in phosphate-buffered saline (PBS)- or HEPES (﻿4-(2-hydroxyethyl)-1-piperazineethanesulfonic acid)-buffered saline (HBS)-treated diabetic mice compared with age-matched controls (Figs. 2 and 3). Dose-dependent experiments revealed that ESC at a concentration of 3 × 10^5^ cells/20 µL partially restored erectile function, which reached up to 78% (maximal ICP) or 76% (total ICP) of control values (Fig. 2). In contrast, intracavernous injection of ESC-NVs restored erectile function in a dose dependent manner in diabetic mice, showing almost complete recovery of erection parameters at a concentration of 1.0 µg/20 µL, which reached up to 95% (maximal ICP) or 97% (total ICP) of normal value (Fig. 3). ESC-NVs (1.0 µg/20 µL) demonstrated superior erectile function recovery than ESC (3 × 10^5^ cells/20 µL) (see Supplemental Fig. 1). No detectable differences in MSBP were found among the experimental groups (Tables 1 and 2).

### ESC-NVs promote angiogenesis under diabetic conditions

**Figure 4:**
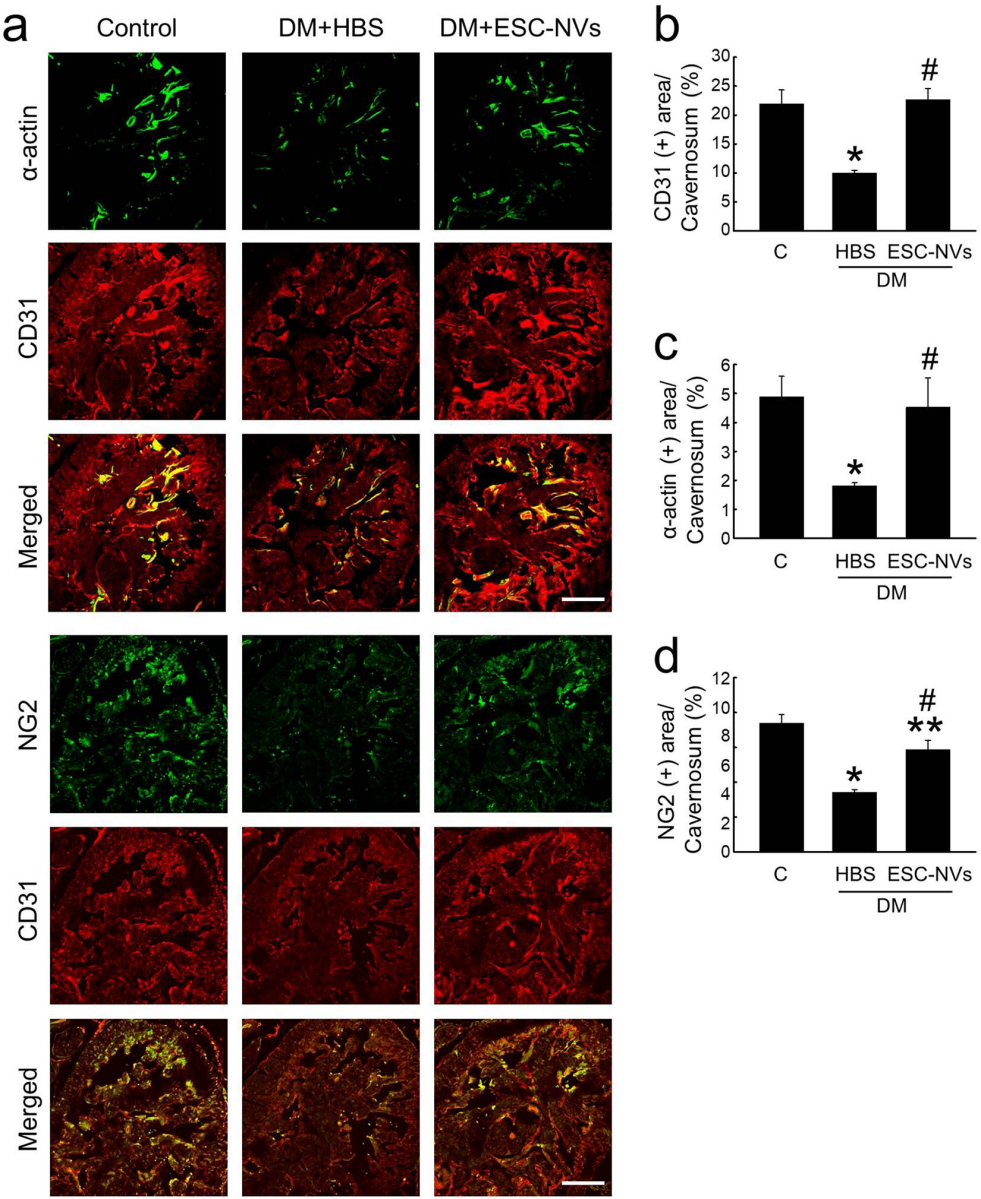
Embryonic stem cell (ESC)-derived extracellular vesicle-mimetic nanovesicles (ESC-NVs) restore cavernous endothelial cell, smooth muscle cell, and pericyte content in the diabetic mice. (**a)** α-acin (green) and PECAM-1 (red) or NG2 (green) and PECAM-1 (red) staining in cavernous tissue from age-matched control (C) and diabetic mice stimulated at 2 weeks after intracavernous injections of HBS (days −3 and 0; 20 μL) or ESC-NVs (days −3 and 0; 1 µg/20 µL). Scale bar = 100 μm. **(b–d)** Quantitative analysis of cavernous endothelial cell, smooth muscle cell, and pericyte content was performed by an image analyzer. Each bar depicts the mean (±SE) values from N = 6 animals per group. **(b)** *P < 0.01 vs. control group; ^#^*P* < 0.01 vs. HBS-treated diabetic group. (**c**) *P < 0.05 vs. control group; ^#^*P* < 0.05 vs. HBS-treated diabetic group. (**d**) *P < 0.01, **P < 0.05, vs. control group**;** ^#^*P* < 0.01 vs. HBS-treated diabetic group. DM, diabetes mellitus; HBS, HEPES (4-(2-hydroxyethyl)-1-piperazineethanesulfonic acid)-buffered saline.

We performed immunofluorescent staining with antibodies against smooth muscle α-actin, platelet/endothelial adhesion molecule 1 (PECAM-1), and neuron-glial antigen 2 (NG2) in the cavernous tissue of control and diabetic mice 2 weeks after treatment of ESC-NVs (1.0 µg/20 µL). The cavernous expression of smooth muscle cell, endothelial cell, and pericyte contents was significantly lower in the HBS-treated diabetic mice than in the control mice. Intracavernous injection of ESC-NVs induced complete restoration of smooth muscle cell and endothelial cell contents, and partial restoration of pericyte content in the diabetic mice (Fig. 4).

**Figure 5:**
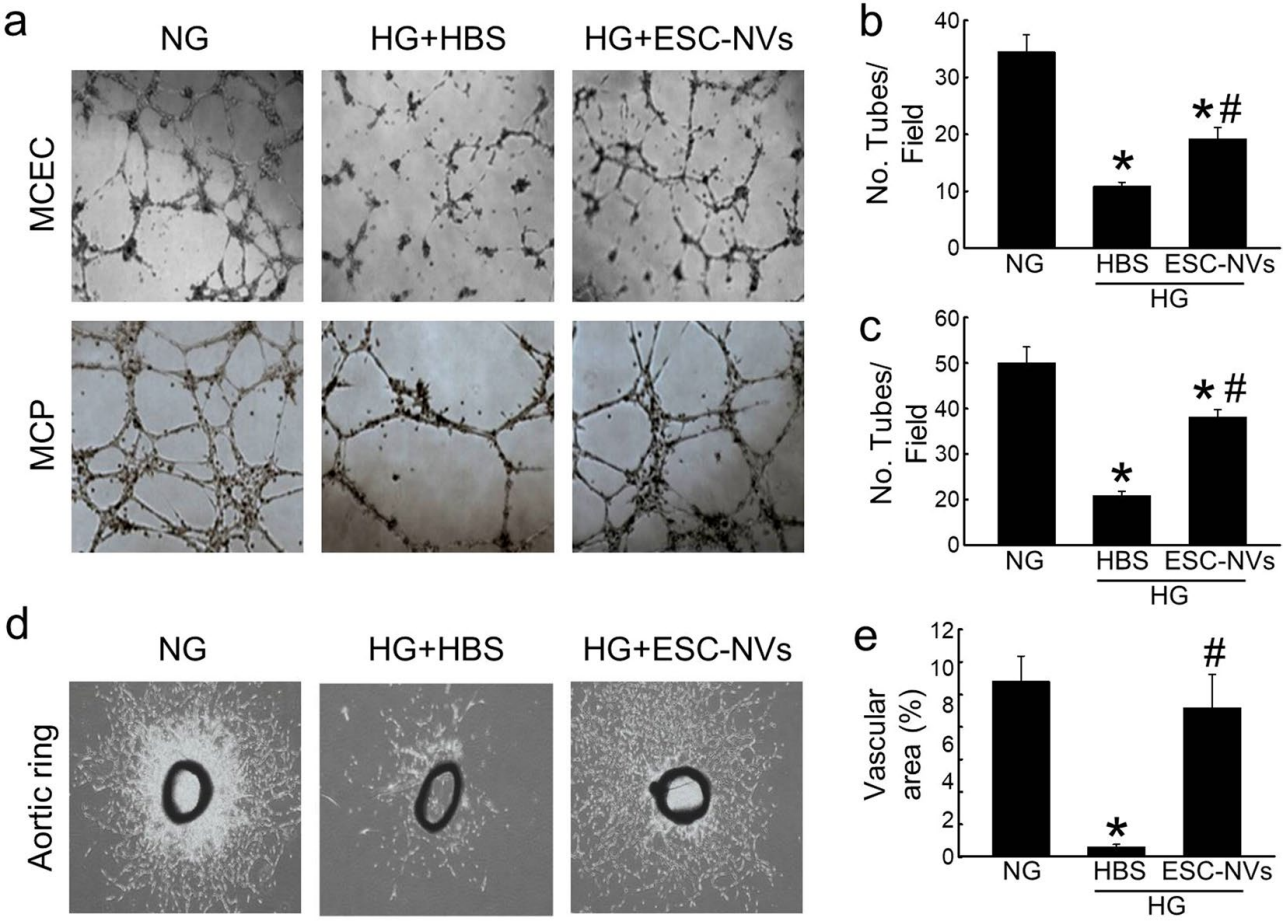
Embryonic stem cell (ESC)-derived extracellular vesicle-mimetic nanovesicles (ESC-NVs) enhance tube formation and microvascular sprouting under diabetic conditions. (**a)** Tube formation assay in mouse cavernous endothelial cell (MCEC) or mouse cavernous pericyte (MCP) exposed to normal-glucose (NG) or high-glucose (HG) conditions for 48 hours and treated with HBS or ESC-NVs (1 µg/mL). 100× magnification. **(b)** *Ex vivo* aortic ring assay. 40× magnification. **(c**,**d)** Number of tubes per high-power field (N = 4). **(c)** *P < 0.01 vs. NG group; ^#^*P* < 0.05 vs. HG + HBS group. (**d**) *P < 0.01 vs. NG group**;** ^#^*P* < 0.01 vs. HG + HBS group. (**e**) Area of outgrowing microvessels from aortic ring (N = 4). *P < 0.01 vs. NG group; ^#^*P* < 0.01 vs. HG + HBS group. HBS, HEPES (4-(2-hydroxyethyl)-1-piperazineethanesulfonic acid)-buffered saline.

An *in vitro* matrigel assay revealed impairments in tube formation in MCEC or MCP exposed to high-glucose condition, and these impairments were partially restored by treatment with ESC-NVs (Fig. 5a–c). An *ex vivo* aortic ring assay revealed significantly decreases in the average length and branch number of outgrowing microvessels in aortic segments exposed to high-glucose condition compared with that in the segments exposed to normal-glucose condition. ESC-NVs significantly enhanced the outgrowth of microvessels from aortic rings under high-glucose condition (Fig. 5d,e).

### ESC-NVs induce neural regeneration under diabetic conditions

**Figure 6:**
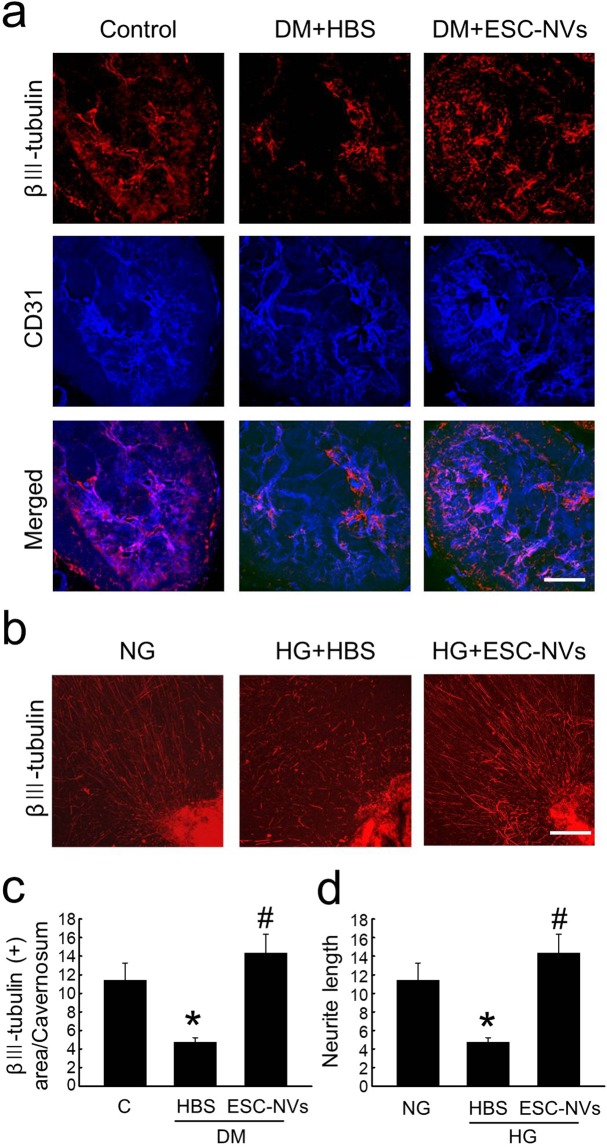
Embryonic stem cell (ESC)-derived extracellular vesicle-mimetic nanovesicles (ESC-NVs) induce neural regeneration under diabetic conditions. (**a)** βIII tubulin (red) and PECAM-1 (blue) staining in cavernous tissue from age-matched control (C) and diabetic mice stimulated at 2 weeks after intracavernous injections of HBS (days -3 and 0; 20 μL) or ESC-NVs (days −3 and 0; 1 µg/20 µL). Scale bar = 100 μm. **(b)** βIII tubulin (red) staining in mouse major pelvic ganglion (MPG) tissue exposed to normal-glucose (NG) or high-glucose (HG) conditions for 72 hours and treated with HBS or ESC-NVs (1 µg/mL). Scale bar = 100 μm. **(c)** Quantitative analysis of βIII tubulin immunopositive areas in cavernous tissue content was performed by an image analyzer. Each bar depicts the mean (±SE) values from N = 6 animals per group. *P < 0.05 vs. control group; ^#^*P* < 0.05 vs. HBS-treated diabetic group. (**d**) Quantification of neurite length was performed by an image analyzer (N = 4). *P < 0.05 vs. NG group; ^#^*P* < 0.01 vs. HG + HBS group. DM, diabetes mellitus; HBS, HEPES (4-(2-hydroxyethyl)-1-piperazineethanesulfonic acid)-buffered saline.

The expression of βIII tubulin in corpus cavernosum was significantly lower in the HBS-treated diabetic mice than in the control mice, whereas the neuronal cell content was completely restored by treatment with ESC-NVs (Fig. 6a,c). ESC-NVs also profoundly enhanced neurite sprouting in an *ex vivo* cultured MPG tissue exposed to high-glucose condition (Fig. 6b,d).

### ESC-NVs increase the expression of angiogenic and neurotrophic factors, and enhance cell proliferative and survival pathway

**Figure 7:**
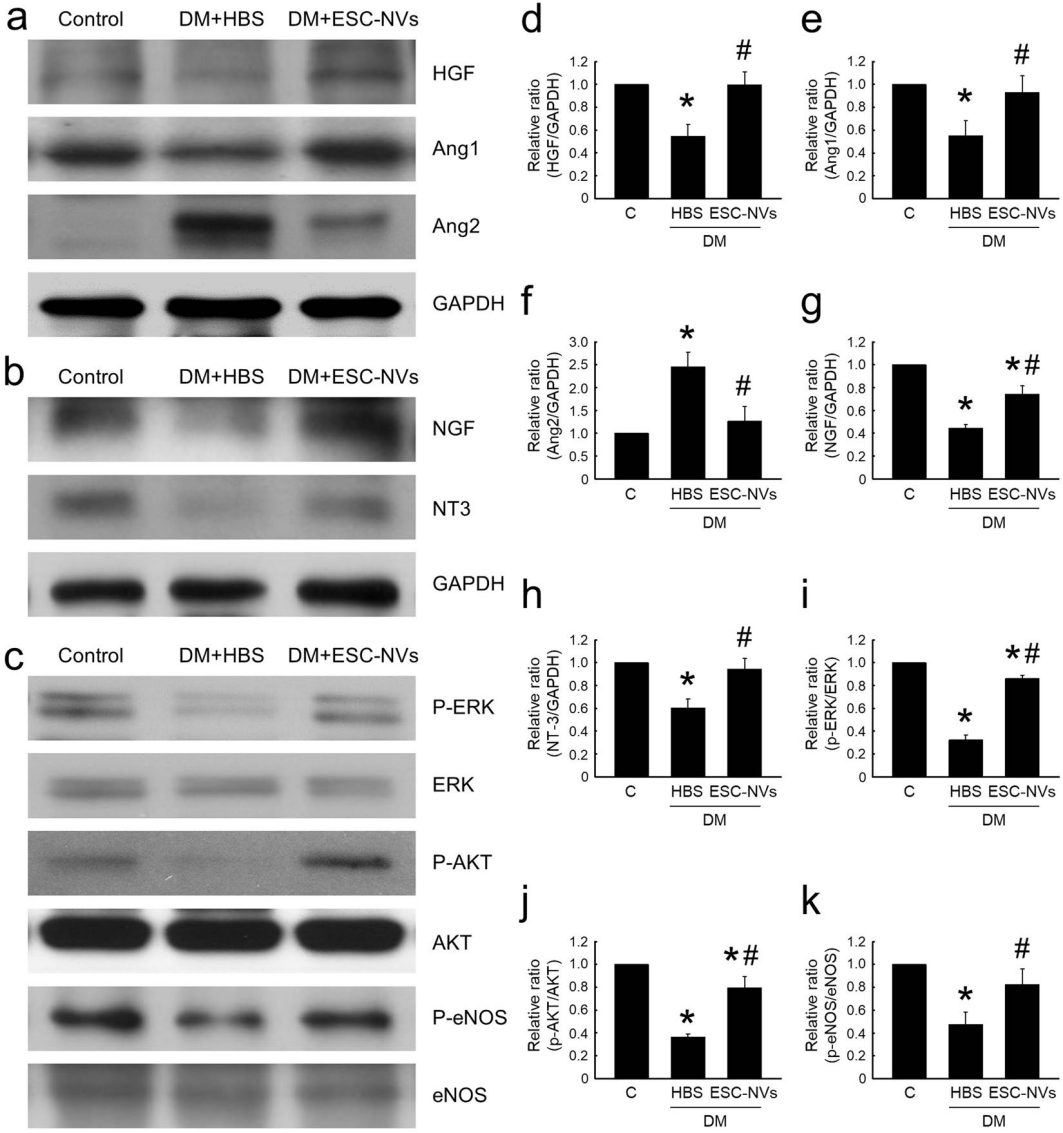
Embryonic stem cell (ESC)-derived extracellular vesicle-mimetic nanovesicles (ESC-NVs) increase the expression of angiogenenic and neurotrophic factors, and induces cell proliferative and survival signaling pathway. (**a)** Representative Western blot for angiogenic factors (hepatocyte growth factor [HGF], angiopoietin-1 [Ang1], and angiopoietin-2 [Ang2]) in penis tissue from age-matched control (C) and diabetic mice stimulated at 2 weeks after intracavernous injections of HBS (days −3 and 0; 20 μL) or ESC-NVs (days −3 and 0; 1 µg/20 µL). **(b)** Representative Western blot for neurotrophic factors (nerve growth factor [NGF] and neurotrophin-3 [NT3]). **(c)** Representative Western blot for cell proliferative and survival factors (phospho-ERK [P-ERK]/ERK, phospho-Akt [P-Akt]/Akt, and phospho-eNOS [P-eNOS]/eNOS). **(d–k)** Normalized band intensity values. Each bar depicts the mean (±SE) values from N = 4 animals per group. The relative ratio in the control group was arbitrarily set to 1. **(d**–**f,h,k)** *P < 0.05 vs. control group; ^#^*P* < 0.05 vs. HBS-treated diabetic group. (**g**,**i**,**j**) *P < 0.05 vs. control group; ^#^*P* < 0.05 vs. HBS-treated diabetic group. DM, diabetes mellitus; HBS, HEPES (﻿4-(2-hydroxyethyl)-1-piperazineethanesulfonic acid)-buffered saline.

Western blot analysis showed that the cavernous expression of hepatocyte growth factor (HGF) and angiopoietin-1 (Ang1) protein was significantly lower and angiopoietin-2 (Ang2) protein expression was significantly higher in the HBS-treated diabetic mice than in the age-matched controls. The expressions of these angiogenic factors were returned to control values after treatment with ESC-NVs (Fig. 7a,d–f).

To test whether the effects of ESC-NVs was mediated by the production of neurotrophic factors, we performed Western blot analysis for nerve growth factor (NGF) and neurotrophin-3 (NT-3). The cavernous expression of NGF and NT-3 was significantly lower in the HBS-treated diabetic mice than in the control mice. ESC-NVs restored the cavernous expression of NGF and NT-3 in the diabetic mice (Fig. 7b,g,h).

Moreover, ESC-NVs also induced phosphorylation of ERK, Akt, and endothelial nitric oxide synthase (eNOS) in the corpus cavernosum of diabetic mice (Fig. 7c,i–k).

## Discussion

Here, we examined the efficacy of ESC-NVs in a mouse model of diabetic ED. Intracavernous administration of ESC-NVs induced almost complete recovery of erectile function in the diabetic mice, whereas intracavernous injection of ESC partially restored erectile function. The beneficial effects of ESC-NVs were accomplished by restoring cavernous contents of endothelial cells, smooth muscle cells, pericytes, and neuronal cells in the diabetic mice *in vivo*; by promoting tube formation in primary cultured MCEC and MCP under high-glucose condition *in vitro*; and by accelerating microvascular and neurite sprouting from MPG under high-glucose condition *ex vivo*, respectively. ESC-NVs induced the expression of angiogenic and neurotrophic factors, and activated cell survival and proliferative factors in the diabetic mice *in vivo*.

Although the EVs do not have a potential of malignant transformation, their proangiogenic and proliferative effects may accelerate cancer progression. Moreover, previous study reported that low dose of endothelial cell-derived EVs increased tube formation, whereas a high concentration had an inhibitory effect^20^. Therefore, it is particularly important to determine optimal dose of EVs to minimize side effects, while enhancing their therapeutic efficacy. In the present study, we determined optimal dosage of ESC-NVs (0.1 μg, 0.5 μg, 1 μg, 2 μg, or 5 μg in 20 μL of HBS, respectively) and obtained a maximal erectile function recovery at a concentration of 1 μg/20 μL.

It was demonstrated that EVs released by endothelial cells contain β1 integrin and matrix metalloproteinase-2 and −9, and promoted endothelial cell invasion and capillary-like tube formation^21^. In the present study, ESC-NVs increased the cavernous expression of Ang1 and HGF, and decreased the expression of Ang2 in diabetic mice. Ang1 is a secreted protein ligand for tyrosine kinase with immunoglobulin and epidermal growth factor homology domain-2 (Tie2, also called Tek). Ang1 has a major role in blood vessel remodeling, maturation, and stabilization^22,23^. Ang2 is an endogenous antagonist of Ang1^24^. We recently reported in mouse models of type I and type II diabetic ED that intracavernous administration of synthetic Ang1 restores erectile function by enhancing endothelial cell regeneration^25,26^. HGF is also known as a potent angiogenic factor that induces migration of endothelial cells and pericytes^27^. We found that recombinant human-HGF protein completely restored cavernous endothelial cell and pericyte contents, and decreased oxidized LDL leakage in the diabetic mice^3,27^. Therefore, the regulation of these angiogenic factors may be an important mechanism by which ESC-NVs enhance angiogenesis under diabetic conditions.

After peripheral nerve injury, Schwann cells are known to release cytokines and secrete neurotrophic factors that guide neural regeneration^28,29^. It was reported that Schwann cell NVs, but not fibroblast NVs, specifically enhanced neurite growth *in vitro*^15^. In this regard, the induction of neurotrophic factors (NGF and NT-3) and subsequent neurite sprouting from *ex vivo* cultured MPG under high-glucose condition as well as axonal regeneration in the diabetic mice *in vivo* by ESC-NVs are noteworthy. However, it remains to clarify the sources of growth factors whether these neurotrophic factors as well as angiogenic factors are directly derived from ESC-NV cargo, or endogenously synthesized secondarily from penile neurovascular regeneration.

Akt is a serine/threonine kinase and downstream signaling mediator of phosphatidylinositol 3-kinase (PI3K), and the activation of PI3K/Akt pathway is known to enhance survival of the various cell types^30^. Activation of ERK pathway is reported to enhance cell proliferation^31,32^. EVs generated in response to interleukin-3 stimulation are known to increase ERK activation and cyclin D1 transcription, and to promote angiogenesis^33^. Moreover, endothelial colony-forming cell-derived EVs enhanced neovascularization and promoted cutaneous wound healing in diabetic rats by activating ERK signaling in endothelial cells and by stimulating the expression of angiogenic molecules^34,35^. EVs isolated from Akt-overexpressing mesenchymal stem cells are also known to stimulate endothelial cell migration, proliferation, and tube-like formation *in vitro*^36^, and to increase the formation of blood vessel *in vivo*. In the present study, ESC-NVs enhanced phosphorylation in endothelial cell survival through the PI3K/Akt pathway^23^ and to induce endothelial cell proliferation by activation of the ERK pathway^23,31,32^. From these findings, we believe that ESC-NV-mediated increase in angiogenic factors in the corpus cavernosum of diabetic mice may play a key role in activating signaling pathway involved in cell survival and proliferation.

We used the most potent stem cell source, ESC, to isolate EV-mimetic NVs and to confirm the efficacy of ESC-NVs in diabetic ED, although ethical concerns might be raised when those are applicable in clinical situation. The cellular or organ source of EVs is also reported to be of great importance, as shown by the *in vivo* tracking study which demonstrated that intravenously administered EVs derived from kidney embryonic cells are taken up mainly by the kidney^37^. Thus, it will be intriguing to compare the results of this study with those of future studies using EVs or EV-mimetic NVs derived from a variety of cells, such as endothelial cells, smooth muscle cells, or pericytes isolated from orthotopic organ, i.e., erectile tissue.

The functionality of EVs is strongly influenced by the microenvironment^38^ or cytokine stimulation^33,39^. For example, hypoxia stimulation and preconditioning of stem cells with platelet-derived growth factor or an endothelial differentiation medium favored the release of EVs with vasculogenic potential and enhanced their proangiogenic activity^33,38,39^. Therefore, it will be valuable to evaluate whether the several stimuli or modification of culture conditions would result in better outcomes.

Our study has some limitations. We did not screen for the development of ED before treatment of ESC-NVs because of invasive nature of the nerve-induced erectile function study. We demonstrated a short-term efficacy of ESC-NVs in a mouse model of diabetic ED. Further studies are needed to test whether ESC-NVs would induce durable erectile function recovery in a variety of animal models for ED.

## Conclusions

Our study demonstrates a unique function of ESC-NVs in the diabetic ED. ESC-NVs ameliorates erectile function in diabetic mice by enhancing penile neurovascular regeneration and demonstrates superior effects than ESC. Local treatment with EV-mimetic NVs may represent a promising therapeutic strategy for the treatment of ED caused by vascular and neural diseases.

## Materials and Methods

### Preparation and characterization of exosome

#### ESC culture

Mouse ESC were maintained on irradiated mouse embryonic fibroblasts in Dulbecco modified Eagle medium (DMEM) (Gibco, Carlsbad, CA, USA) with 15% fetal bovine serum (Gibco), 1000 U/mL LIF (Chemicon International, Temecula, CA, USA), 100 U/mL penicillin/streptomycin (Invitrogen, Corp., Carlsbad, CA, USA), L-Glutamine 200 mM (100×) (Gibco), 0.1 mM nonessential amino acids (Gibco), and 0.1 mM β-mercaptoethanol (Gibco) at 37 °C/5% CO_2_. Media was changed daily, and cells were passaged every 2 to 3 days.

#### Preparation of ESC-NVs

ESC-NVs were prepared as described previously^19^. Briefly, mouse ESC were rinsed with PBS. Adherent cells were detached using 0.25% Trypsin-EDTA (Invitrogen) and re-suspended in HEPES buffer solution (Gibco). *ESC-NVs* were produced using a mini extruder system (Avanti Polar Lipids, Birmingham, AL, USA). Cell suspension was sequentially extruded using 10, 5, and 1 μm pore-sized polycarbonate membrane (Nuclepore, Whatman Inc., Clifton, NJ, USA) ten times across each filters. To form a step gradient, 50% iodixanol (1 mL; Axis-Shield PoC AS, Oslo, Norway) was placed at the bottom of an ultracentrifuge tube, overlaid with 10% iodixanol (2 mL) and the extruded samples (7 mL), and then ultracentrifuged at 100,000 g for 2 hours at 4 °C. The interface layers between the 10% and 50% iodixanol were further pelleted at 100,000 g for 2 hours at 4 °C. NVs were filtered with 0.45 μm filter and stored at −80 °C until use (Fig. 1a).

#### Transmission electron microscopy

The purified ESC-NVs were applied to glow-discharged carbon-coated copper grids (Electron Microscopy Sciences, Fort Washington, PA, USA). After ESC-NVs had been allowed to be absorbed onto the grid for 1 hour, the grids were fixed with 4% paraformaldehyde for 10 minutes and rinsed with droplets of deionized water and then, negatively stained with 2% uranyl acetate (Ted Pella, Redding, CA, USA). Electron micrographs were recorded with a JEM 1011 microscope (JEOL, Tokyo, Japan) at an acceleration voltage of 100 kV as described previously^19^.

#### Dynamic light scattering

The size distribution of ESC-NVs was measured with Zetasizer Nano ZS (Malvern Instrument Ltd., Malvern, U.K.). The size distribution based on relative abundance was determined by an infrared light (wavelength = 633 nm) passing through the sample at the scattered intensity of 10× for 30 seconds as described previously^19^.

#### Western blot analysis

ESC-NVs and whole cell lysates were separated by SDS-PAGE (10% resolving gel), and then transferred to a polyvinylidene difluoride membrane. Each blot was blocked, and probed with antibodies to GM130 (BD Biosciences, San Jose, CA, USA; 1:1000), CD63 (Novus Biologicals, Liggleton, CO, USA; 1:1000), CD81 (Novus Biologicals; 1:1000), or TSG101 (Novus Biologicals; 1:500).

### Animals and treatments

Eight-week-old male C57BL/6 mice were used in this study. The experiments were approved by the Institutional Animal Care and Use Committee of Inha University (Assurance Number: INHA 171129-527) and performed in accordance with relevant guidelines and regulations. Diabetes was induced by intraperitoneal injection of multiple low doses of streptozotocin (STZ, 50 mg/kg body weight in 0.1 M citrate buffer, pH 4.5) for 5 consecutive days as described previously^40^. Animals were considered diabetic if their nonfasting glucose levels were greater than 300 mg/dL. Eight weeks after diabetes was induced, the mice were anesthetized with intramuscular injections of ketamine (100 mg/kg) and xylazine (5 mg/kg) and placed supine on a thermoregulated surgical table.

To test the efficacy of ESC, the mice were distributed into six groups (N = 5 per group): age-matched controls and STZ-induced diabetic mice receiving repeated intracavernous injections of PBS (days −3 and 0; 20 μL) or ESC (days −3 and 0; 1 × 10^4^ cells, 1 × 10^5^ cells, 3 × 10^4^ cells, or 1 × 10^6^ cells in 20 μL of PBS, respectively).

To test the efficacy of ESC-NVs, the mice were distributed into seven groups (N = 5 per group): age-matched controls and STZ-induced diabetic mice receiving repeated intracavernous injections of HBS (days −3 and 0; 20 μL) or ESC-NVs (days −3 and 0; 0.1 μg, 0.5 μg, 1 μg, 2 μg, or 5 μg in 20 μL of PBS, respectively). ESC-NVs were given twice, because our pilot experiments demonstrated that a single intracavernous injection of ESC-NVs resulted in a partial recovery of erectile function (data not shown). To minimize leakage of the ESC or ESC-NVs into systemic circulation, blood drainage via the dorsal veins was halted by circumferential compression of the penis at the base with an elastic band immediately before injection, and the compression was released at 30 minutes after the injection^41^. We evaluated erectile function by cavernous nerve electrical stimulation 2 weeks after treatment. A separate group of animals was used for histologic examination and biochemical study.

### Measurement of erectile function

The mice from each group were anesthetized with ketamine (100 mg/kg) and xylazine (5 mg/kg) intramuscularly. Erectile function was measured as described previously^40^. Briefly, the bladder and prostate were exposed through a midline abdominal incision. The MPG and cavernous nerve were identified posterolaterally to the prostate on one side, and bipolar platinum wire electrodes were placed around the cavernous nerve for electrical stimulation. The penis was denuded of skin, and a 26-gauge needle filled with 250 U/mL of heparin was inserted into one side of the corpus cavernosum for monitoring ICP with a Statham P23 pressure transducer connected to a computerized system for data acquisition (Biopac Systems, Goleta, CA, USA), which was interfaced to a personal computer for recording and data analysis. Stimulation parameters were 5 V at a frequency of 12 Hz, a pulse width of 1 ms, and a duration of 1 minute. During tumescence, the maximal ICP was recorded. The total ICP was determined by the area under the curve from the beginning of cavernous nerve stimulation to a point 20 seconds after stimulus termination. Systemic blood pressure was measured by using a noninvasive tail-cuff system (Visitech systems, Apex, NC, USA). The ratios of maximal ICP and total ICP (area under the curve) to MSBP were calculated to adjust for variations in systemic blood pressure.

### Tube formation assay

The MCEC and MCP were prepared and maintained as described previously^3^. The tube formation assay was performed to assess the angiogenic capacity of ESC-NVs in MCEC or MCP. About 100 µL of growth factor-reduced matrigel was dispensed into 96-well tissue culture plates at 4 °C. After gelling at 37 °C for at least 30 minutes, the MCEC or MCP were seeded onto the gel at 4 × 10^4^ cells/well in 200 µL of M199 or DMEM medium. The assay was performed in a CO_2_ incubator and the plates were incubated at 37 °C for 24 hours. Images were obtained with a phase-contrast microscope and the numbers of tubes in each well of the plate were counted at a screen magnification of ×40 (N = 4 per group). Only integrated tubes were counted.

### Aortic ring assay

Aortic ring assay were performed as described previously^42^. Aortas were harvested from 8-week-old C57BL/6 mice (N = 4 per group). The aortic rings were placed in the 8-well Nunc Lab-Tek Chamber Slide System (Sigma-Aldrich, Saint Louis, MO, USA) and sealed in place with an overlay of 50 μL matrigel. The aortic rings were cultured in medium 199 with 20 ng/mL of basic fibroblast growth factor and 1% penicillin/streptomycin for 5 days. The aortic segments and sprouting cells were fixed in 4% paraformaldehyde for at least 30 minutes and used for immunofluorescent staining.

### *Ex Vivo* neurite sprouting assay

The mouse major pelvic ganglion tissues were prepared and maintained as described previously^43^ with minor modifications. The MPG tissues were isolated from male mice using a microscope, transferred into sterile vials containing Hank’s balanced salt solution (Gibco), and then rinsed and washed twice in PBS. The MPG tissues were cut into small pieces and the samples plated on poly-D-lysine hydrobromide-coated (Sigma-Aldrich) 12-well plate. The whole MPG tissue was covered with matrigel and the culture plate placed on ice for 5 minutes prior to incubation at 37 °C for 10–15 minutes in a 5% CO_2_ atmosphere. We added 1 mL of complete Neurobasal medium (Gibco) supplemented with 2% serum-free B-27 (Gibco) and 0.5 nM GlutaMAX™-I (Gibco). The dishes were then incubated at 37 °C in a 5% CO_2_ atmosphere. Three days after incubation, we evaluated neurite outgrowth.

### Establishment of *In Vitro* or *Ex Vivo* experimental systems that mimic diabetic ED

To mimic an *in vivo* or *ex vivo* condition for diabetes-induced angiopathy and neuropathy, primary cultured cells or tissues were serum-starved for 24 hours and then exposed to normal-glucose (5 mmol) or high-glucose (30 mmol; Sigma-Aldrich) conditions for 2 days (MCEC and MCP), 3 days (MPG tissue), or 5 days (aortic ring) as described previously^42^.

### Histological examinations

The penis tissue (N = 6 per group) and cultured MPG (N = 4 per group) were fixed in 4% paraformaldehyde for 24 hours at 4 °C as described previously^42^. Frozen tissue sections (12-μm or 60-μm thick) were incubated with antibodies to smooth muscle α-actin (Sigma-Aldrich; 1:50), NG2 (Millipore, Temecula, CA, USA; 1:50), PECAM-1 (Millipore; 1:50), or βIII tubulin (Abcam, Cambridge, UK; 1:50) at 4 °C overnight. After several washes with PBS, the tissues were incubated with tetramethyl rhodamine isothiocyanate- or fluorescein isothiocyanate-conjugated secondary antibodies (Zymed Laboratories, South San Francisco, CA, USA) for 2 hours at room temperature. Samples were mounted. Signals were visualized and digital images were obtained with a confocal microscope (FV1000, Olympus, Tokyo, Japan). Quantitative analysis of histologic examinations was done with an image analyzer system (National Institutes of Health [NIH] Image J 1.34, http://rsbweb.nih.gov/ij/) and we analyzed the histologic data in a blinded manner.

### Western blot analysis

Equal amounts of protein (40 µg per lane) were electrophoresed on sodium dodecylsulfate-polyacrylamide gels (8% to 15%), transferred to polyvinylidene difluoride membrane, and probed with antibodies to HGF (Santa Cruz Biotechnology, Delaware CA, USA; 1:1000), Ang1 (Novus Biologicals), Ang2 (Novus Biologicals; 1:1000), phospho-eNOS (Signaling, Beverly, MA, USA; 1:250), eNOS (Cell Signaling; 1:500), phospho-Akt (Cell Signaling; 1:1000), Akt (Cell Signaling; 1:1000), phospho-ERK (Cell Signaling; 1:1000), ERK (Cell Signaling; 1:1000), NGF (Santa Cruz Biotechnology; 1:1000), NT-3 (Santa Cruz Biotechnology; 1:1000), GAPDH (ABclonal, Woburn, MA, USA; 1:5000), or β-actin (Abcam; 1:6000). The results were quantified by densitometry (N = 4 per group).

### Statistical analysis

The results are expressed as mean ± SE. For parametric data, intergroup comparisons were made by one-way ANOVA followed by Newman-Keuls posthoc tests. We used the Kruskal-Wallis test to compare nonparametric data. Probability values less than 5% were considered significant. We used SigmaStat 3.11 software (Systat Software) for statistical analyses. We also performed a power analysis to determine the minimum number of animals that need to be included in the present study (https://clincalc.com/stats/samplesize.aspx).

## Supplementary information

Supplementary Info
