# Supplementary material for: Embryonic stem cell-derived extracellular vesicle-mimetic nanovesicles rescue erectile function by enhancing penile neurovascular regeneration in the streptozotocin-induced diabetic mouse
Source: Sci Rep. 2019 Dec 27;9:20072. doi: 10.1038/s41598-019-54431-4 (PMC6934510; doi:10.1038/s41598-019-54431-4)

**Embryonic stem cell-derived extracellular vesicle-mimetic nanovesicles rescue erectile function by enhancing penile neurovascular regeneration in the streptozotocin-induced diabetic mouse**

Mi-Hye Kwon^1,*^, Kang-Moon Song^1,*^, Anita Limanjaya^1^, Min-Ji Choi^1^, Kalyan Ghatak^1^,

Nguyen Nhat Minh^1^, Jiyeon Ock^1^, Guo Nan Yin^1^, Ju-Hee Kang^2^, Man Ryul Lee^3^,

Yong Song Gho^4^, Ji-Kan Ryu^1,†^, and Jun-Kyu Suh^1, †^

^1^National Research Center for Sexual Medicine and Department of Urology, Inha University School of Medicine, Incheon 22332, Korea

^2^Department of Pharmacology and Medicinal Toxicology Research Center, Inha University School of Medicine, Incheon 22212, Korea

^3^Soonchunhyang Institute of Medi-bio Science (SIMS) and Institute of Tissue Regeneration, College of Medicine, Soon Chun Hyang University, Cheonan-si, Chungcheongnam-do 31151, Korea

^4^Department of Life Sciences, Pohang University of Science and Technology, Pohang, Kyeongsangbuk-do 37673, Korea

^*^These authors contributed equally to this study.

^†^Correspondence to Jun-Kyu Suh, MD, PhD and Ji-Kan Ryu, MD, PhD

Jun-Kyu Suh, MD, PhD

National Research Center for Sexual Medicine and Department of Urology

Inha University School of Medicine

27, Inhang-Ro, Jung-Gu, Incheon 22332

Republic of Korea

Tel: 82-32-890-3441, Fax: 82-32-890-3097

E-mail: jksuh@inha.ac.kr

Ji-Kan Ryu, MD, PhD

National Research Center for Sexual Medicine and Department of Urology

Inha University School of Medicine

27, Inhang-Ro, Jung-Gu, Incheon 22332

Republic of Korea

Tel: 82-32-890-3505; Fax: 82-32-890-3099

E-mail: rjk0929@inha.ac.kr

**
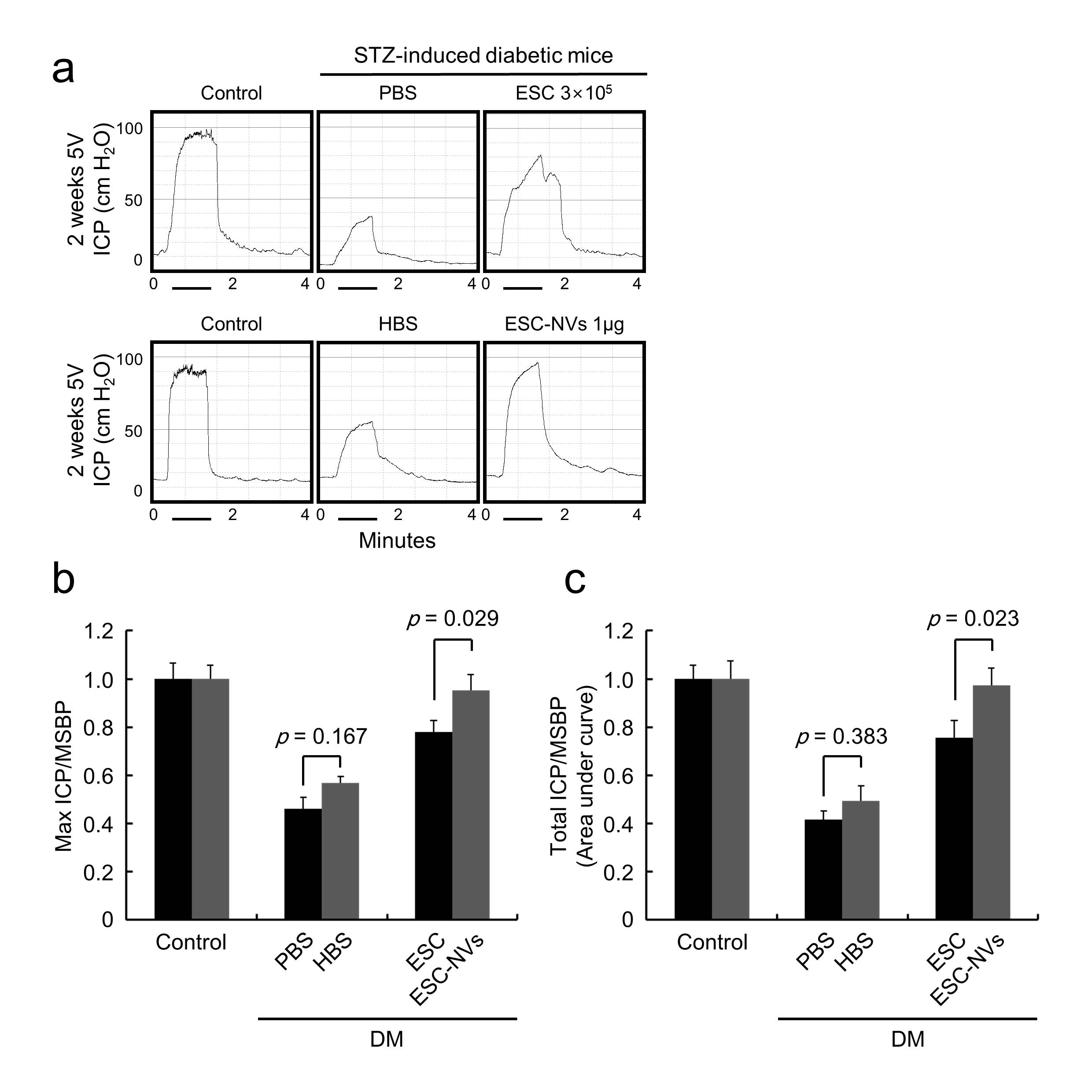
**

**Supplemental Figure 1. Embryonic stem cell (ESC)-derived extracellular vesicle-mimetic nanovesicles (ESC-NVs) fully, and ESC partially restore erectile function in the diabetic mice. (a)** Representative intracavernous pressure (ICP) responses for the age-matched control (C) and diabetic mice stimulated at 2 weeks after intracavernous injections of PBS or HBS (days -3 and 0; 20 μL), and ESC (days -3 and 0; 3 × 10^5^ cells/20 µL) or ESC-NVs (days -3 and 0; 1 µg/20 µL). The stimulus interval is indicated by a solid bar. **(b and c)** Ratios of mean maximal ICP and total ICP (area under the curve) to mean systolic blood pressure (MSBP) were calculated for each group. Each bar depicts the mean (± SE) values from N = 5 animals per group. The values in the control group were arbitrarily set to 1. DM, diabetes mellitus; HBS, HEPES (﻿4-(2-hydroxyethyl)-1-piperazineethanesulfonic acid)-buffered saline; STZ, streptozotocin.

**Abbreviations**

Ang1 = angiopoietin-1; Ang2 = angiopoietin-2; DMEM = Dulbecco modified Eagle medium; ED = erectile dysfunction; eNOS = endothelial nitric oxide synthase; ESC = embryonic stem cell; ESC-NVs = embryonic stem cell-derived extracellular vesicle-mimetic nanovesicles; EVs = extracellular vesicles; HGF = hepatocyte growth factor; ICP = intracavernous pressure; MCEC = mouse cavernous endothelial cell; MCP = mouse cavernous pericyte; MPG = major pelvic ganglion; MSBP = mean systolic blood pressure; NG2 = neuron-glial antigen 2; NGF = nerve growth factor; NO = nitric oxide; NT-3 = neurotrophin-3; NVs = nanovesicles; PDE5 = phosphodiesterase type 5; PECAM-1 = platelet/endothelial adhesion molecule 1; PI3K = phosphatidylinositol 3-kinase; STZ = streptozotocin.


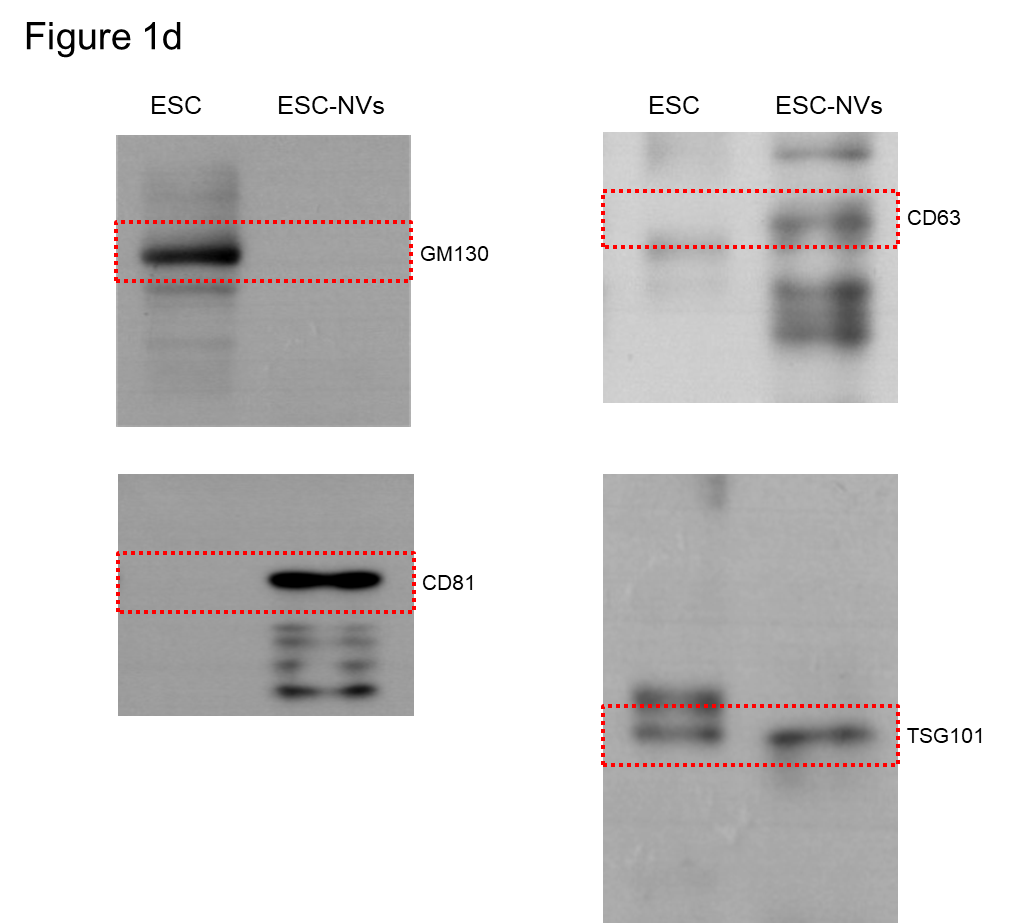
 **Raw data for Western blot**


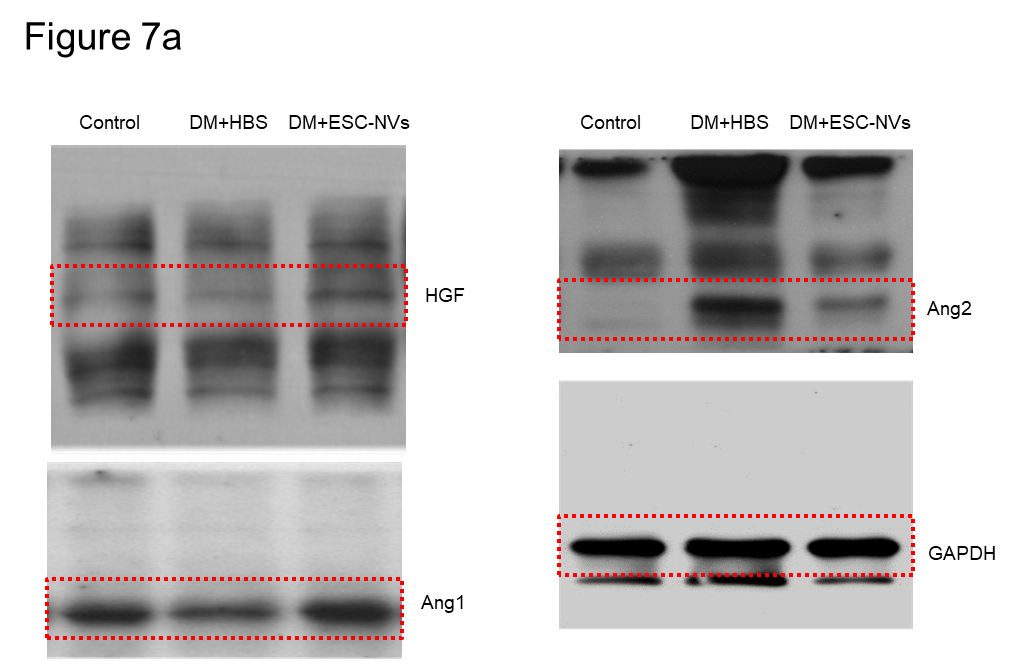


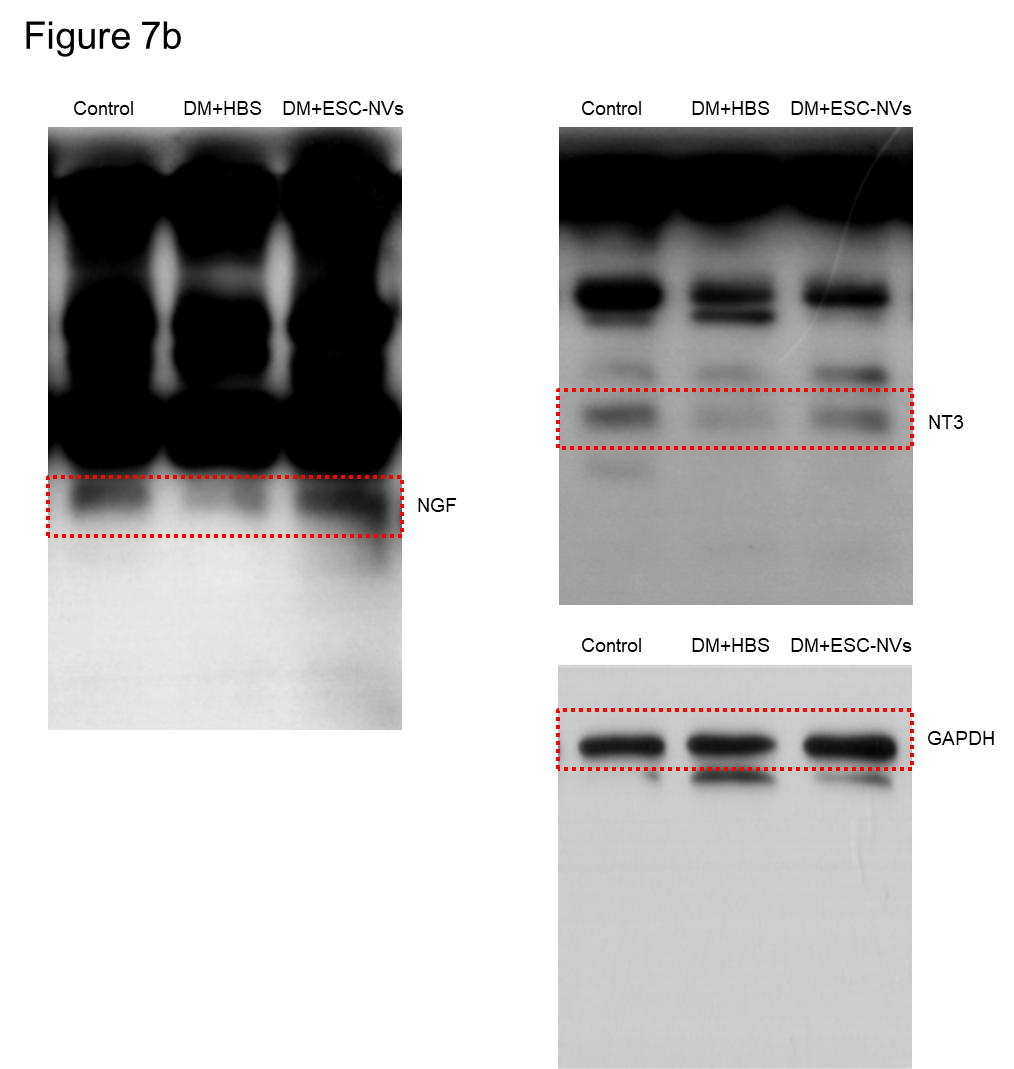


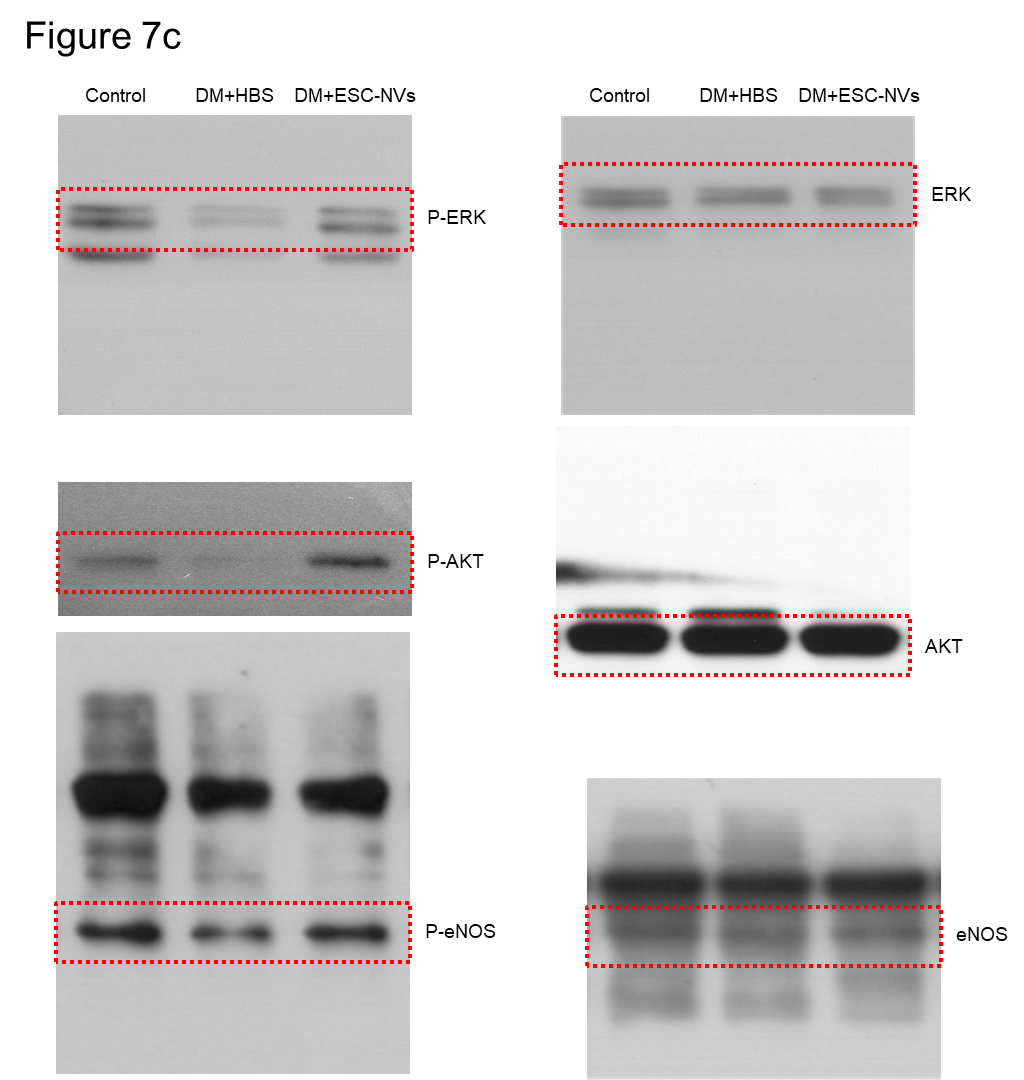

Supplement: Supplementary file 1 — Supplementary Info [file 41598_2019_54431_MOESM1_ESM.docx]
